# Environment-Dependent Genotype-Phenotype Associations in Avian Breeding Time

**DOI:** 10.3389/fgene.2017.00102

**Published:** 2017-08-04

**Authors:** Phillip Gienapp, Veronika N. Laine, A. C. Mateman, Kees van Oers, Marcel E. Visser

**Affiliations:** Department of Animal Ecology, Netherlands Institute of Ecology (NIOO-KNAW) Wageningen, Netherlands

**Keywords:** avian breeding time, genotype-by-environment interaction, GWAS, *Parus major*, phenotypic plasticity, wild population

## Abstract

Understanding how genes shape phenotypes is essential to assess the evolutionary potential of a trait. Identifying the genes underlying quantitative behavioral or life-history traits has, however, proven to be a major challenge. The majority of these traits are phenotypically plastic and different parts of the genome can be involved in shaping the trait under different environmental conditions. These variable genotype-phenotype associations could be one explanation for the limited success of genome-wide association studies in such traits. We here use avian seasonal timing of breeding, a trait that is highly plastic in response to spring temperature, to explore effects of such genotype-by-environment interactions in genome-wide association studies. We genotyped 2045 great tit females for 384081 single nucleotide polymorphisms (SNPs) and recorded their egg-laying dates in the wild. When testing for associations between SNPs and egg-laying dates, no SNP reached genome-wide significance. We then explored whether SNP effects were modified by annual spring temperature by formally testing for an interaction between SNP effect and temperature. The models including the SNP^∗^temperature interaction performed consistently better although no SNP reached genome-wide significance. Our results suggest that the effects of genes shaping seasonal timing depended on annual spring temperature. Such environment-dependent effects are expected for any phenotypically plastic trait. Taking these effects into account will thus improve the success of detecting genes involved in phenotypically plastic traits, thereby leading to a better understanding of their evolutionary potential.

## Introduction

Environmental change, as, e.g., global warming, will inevitably lead to novel selection pressures, especially in phenological traits, as, for example, timing of breeding, migration or hibernation (Gienapp et al., 2014), and in the long run only adaptive microevolution (‘evolutionary rescue’) will enable population persistence (Bürger and Lynch, 1995; Visser, 2008; Gienapp et al., 2013; Gonzalez et al., 2013). Understanding the genetics of traits that are affected by environmental change is important, as this will allow us to assess the adaptive potential of these traits. From quantitative genetic studies we know that many traits, including ecologically relevant phenological, life-history or behavioral traits, are heritable (Mousseau and Roff, 1987), and hence that evolutionary adaptation to climate change would be possible. Heritability is, however, not necessarily constant across environments (Pigliucci, 2001, 2005; Nussey et al., 2007; Husby et al., 2011), which complicates predictions about evolutionary adaptation. This is especially true when environmental conditions will shift beyond the currently observed range due to climate change. Consequently, in the end a better understanding of the genomics of the traits under selection will help us to predict whether they will be able to adapt fast enough to novel environmental conditions.

Due to the advancement in molecular genetics, it is now possible to map the loci that underlie traits of interest in natural populations of ‘ecological model species’ and this has moved the research field of ecological genetics into the genomics era (Slate et al., 2010). So far, however, the number of published mapping studies in natural populations is limited. Over the last decade, over 20 published studies aimed to identify loci underlying almost 40 traits in natural populations (Supplementary Table S1). A number of studies successfully identified some of the loci underlying, for example, wing length in birds, age at maturity in Salmon, or birth weight and recombination rate in mammals (Slate et al., 2002; Tarka et al., 2010; Barson et al., 2015; Johnston et al., 2016). In these studies the identified loci explained moderate to substantial amounts of the genetic variation but in other studies that could identify loci the amount of variation explained by them was (very) small (e.g., Husby et al., 2015; Wenzel et al., 2015). In general, in only about one third of the analyzed traits, one or more loci or genomic regions associated with the trait could be identified. The total number of studies aiming at mapping loci in wild populations that could not identify any locus may even be larger because these studies may simply not have been published due to their non-significant results. There are a number of possible reasons behind this limited success of gene-mapping in wild populations. For example, in some studies the number of individuals was only in the low hundreds. Heritabilities of life-history traits also tend to be low to moderate (Mousseau and Roff, 1987), which would make detecting loci more difficult. Another potential reason for the limited success of these gene-mapping studies in wild populations could be that phenotypic plasticity complicates the detection of relationships between genotype and phenotypes. Many life-history and behavioral traits but also morphological traits are phenotypically plastic (Pigliucci, 2005).

In phenotypically plastic traits the same genotype can produce different phenotypes, and these different phenotypes can hence not be the product of genomic sequence variation alone but must be caused by environmentally induced differential gene expression or post-translational mechanisms (Snell-Rood et al., 2010; Weake and Workman, 2010; Huntzinger and Izaurralde, 2011). This means that the effects of certain genes will be reduced, entirely shut down, or altered under specific environmental conditions. Consider, for example, three genes, A, B and C, affecting a plastic trait: In environment X the expression of gene A may be up-regulated and the expression of gene B down-regulated, while in environment Y the expression of gene A may be down-regulated and the expression of gene B up-regulated. In contrast, the expression of gene C may be neither up- nor downregulated in these environments. A mapping study not taking phenotypic plasticity into account will only identify gene C as associated with the plastic trait because sequence variation in genes A and B is only associated with the trait in part of the data. If post-translational mechanisms are affected, the effect of a gene could not only disappear, due to environmentally dependent downregulation, but its phenotypic effects could even be reversed in one environment compared to the other. Only when modeling an interaction between marker effects and environment the effects of genes A and B will be detected. Such a variable genomic architecture of plastic traits may make detecting loci underlying such traits more difficult if phenotypes are measured in different environment, which is likely to be the case in natural populations, and phenotypic plasticity is not taken into account. Genotype-by-environment interactions have been tested in laboratory or medical studies (Mackay et al., 2009; Thomas, 2010) but not in studies of natural populations where their effects should, however, be even more important.

Breeding time, i.e., the timing of egg-laying, is a highly plastic trait in birds. Depending on spring temperature breeding time can vary by weeks from 1 year to the next (Gienapp et al., 2005, 2010; Charmantier et al., 2008). Besides temperature, photoperiod is known to affect breeding time in birds (Lambrechts et al., 1997; Lambrechts and Perret, 2000) and there is also evidence that the roles of temperature and photoperiod differ in early and late spring (Gienapp et al., 2005, 2010). As pointed out above, phenological traits are expected to come under selection from climate change and increasing spring temperatures have led to mismatches between breeding time and food supply in birds and other species (Visser et al., 1998; Visser and Both, 2005). Understanding the genomics of avian breeding time is hence interesting in the context of climate change, which makes it a very suitable candidate to test whether a variable genomic architecture can obscure genotype-phenotype links. We here use a unique data set consisting of wild 2045 female great tits with recorded egg-laying dates that also have been genotyped on a recently developed 650k SNP chip to test whether the association between SNPs and egg-laying date interacts with spring temperature.

## Materials and Methods

### Phenotypes and Sample Collection

The timing of egg-laying of great tits (*Parus major*) has been studied in our long-term study populations on the ‘Veluwe’ area near Arnhem (52° 00′ N, 5° 50′ E, Netherlands) since the 1950s. Great tits are small, hole-breeding passerine birds that readily accept nest boxes, which has made them a ‘model system’ in behavioral and evolutionary ecology. In our study populations nest boxes are supplied in abundance so that almost the entire population breeds in boxes and can be monitored. Nest boxes were checked weekly in spring and the dates when the first egg of a clutch was laid (from now on: egg-laying date) were back-calculated from the number of recorded eggs assuming one egg is laid per day. All nestlings were banded with standard aluminum bird-bands and blood sampled when seven to 10-days-old. Adult great tits were caught when feeding their chicks with spring-traps in the nest box, identified from their bands, and blood-sampled. Whole blood samples were stored in either 1 ml Cell Lysis Solution (Gentra Puregene Kit, Qiagen, United States) or Queens buffer (Seutin et al., 1991). Field work, including capturing and blood-sampling birds, was carried out under a license of the Animal Experimental Committee of the Royal Dutch Academy of Sciences (KNAW) protocol NIOO-10.07.

### Genotyping of Birds

DNA was extracted from blood samples by using the FavorPrep 96-Well Genomic DNA Extraction Kit (Favorgen Biotech corp.). DNA quality and DNA concentration were measured on a Nanodrop 2000 (Thermo Scientific). A total of 2311 female great tits were genotyped using a custom made Affymetrix^®^ great tit 650K SNP chip at Edinburgh Genomics (Edinburgh, United Kingdom). SNP calling was done following the Affymetrix^®^ best practices workflow by using the Axiom^®^ Analysis Suite 1.1. Eight individuals with dish quality control value of <0.82 were discarded. Dish quality control is an Affymetrix-specific QC measure and we used the default threshold to exclude individual samples. The recommended SNP group (PolyHighResolution, NoMinorHom, MonoHighResolution, CallRateBelowThreshold, Hemizygous) consisted of 537174 SNPs while 73796 SNPs were discarded. In addition to the SNPs that did not pass the quality control steps, an additional 234 SNPs were removed because they were duplicates or the genomic position was missing (NCBI *Parus major* genome version 1.1, GCA_001522545.2). Altogether 536940 SNPs passed initial quality control and 2303 individuals were included for downstream analyses.

### Quality Control of Genotypes

The GenABEL package v1.8-0 (Aulchenko et al., 2007) implemented in R v3.2.0 (R Core Team, 2015) was used to perform a quality control on the dataset. 15719 SNPs and 24 individuals were discarded, because of being monomorphic and/or a low call rate (<95%). The mean autosomal heterozygosity was 0.342 and nine additional individuals were discarded because of a high heterozygosity (FDR < 1%). The mean identity by state of SNPs (IBS) was 0.696 and 14 individuals were discarded because of high IBS value (≥0.95). We did not exclude SNPs that deviated from Hardy-Weinberg equilibrium (HWE) because egg-laying date in our study populations is under directional selection and causal loci may therefore deviate from HWE. We used a MAF-threshold of 0.1 because we wanted to avoid small numbers of individuals for any genotype-temperature class group. We furthermore excluded all SNPs for that not all three genotypes were present and also SNPs that had less than 20 individuals in any genotype-temperature class group.

In PLINK 1.07 (Purcell et al., 2007) we calculated genomic relatedness, based on IBS, for all individuals across all SNPs and a genetic distance from this. This genetic distance matrix was then used, also in PLINK, for multi-dimensional scaling (MDS) to identify whether there was clustering of genetically similar individuals. Twenty-one individuals were identified as outliers and excluded (Supplementary Figure S1). In total 384081 SNPs and 2249 individuals passed the quality control. Given the size of the great tit genome of about 1.02 Gbp (Laine et al., 2015) we have a coverage of roughly 1 SNP per 2700 bp. Of these individuals 2045 had records for at least one egg-laying date and could be included in the analysis.

### Statistical Analyses

Individual egg-laying dates were corrected for among-year and among-area variation prior to analyses by centering to the annual mean egg-laying date separately per area. To test whether SNP effects differed depending on spring temperature we divided the years in our data set into three categories according to their spring temperature. For every year we calculated the average daily mean temperature for the period March 11 to April 20. Average temperature during this period correlates best with annual mean egg-laying dates for our study population (Visser et al., 2006). The used daily mean temperatures from the weather station De Bilt were obtained from the website of the Royal Dutch Meteorological Institute (KNMI^1^). We then calculated the 33%iles and grouped the first, second and third 33%ile into the ‘cold,’ ‘intermediate’ and ‘warm’ category, respectively. The ‘cold’ category comprised 1074 observed egg-laying date of 941 individuals in 6 years, the ‘intermediate’ category 770 observed egg-laying dates of 651 individuals in 6 years and the ‘warm’ category 1615 observed egg-laying dates of 1310 individuals in 5 years. Note that the total number of individuals in our analysis (2045) is not the sum of the individuals breeding in cold, intermediate and warm years, since the same individual can be included in more than one category. The average (and standard deviation in brackets) of the temperature during the period March 11 to April 20 was 6.2°C (0.96°C) for the ‘cold’ category, 7.8°C (0.27°C) for the ‘intermediate’ category, and 9.4°C (0.66°C) for the ‘warm’ category, respectively. Our model to test for SNP effect and their interaction with temperature was:

$$y_{i,j}=\mu +{age}_{i}+{SNP}_{i}+{temp}_{i}:{SNP}_{i}+{pe}_{i}+a_{i}+e_{i,j}$$

where the phenotype *y_i,j_* of observation *j* of individual *i* is a function of the population mean μ, its age (two-level factor: first year breeder or older), its SNP genotype, the interaction between SNP genotype and temperature class *temp*, its ‘permanent environment’ (i.e., individual) effect *pe_i_*, its breeding values *a_i_* and the residual effect *e_i_*. μ, age, the SNP genotype and temperature class are fixed effects and *pe_i_* and *a_i_* random effects. The variance-covariance matrix for *a* is derived from the expected covariance between individuals due to their additive genetic effects. Fitting the additive genetic variance-covariance matrix accounts for the fact that individuals in our study population are related.

All GWA analyses were run in ASReml-R 3.0 (Gilmour et al., 2015). Due to computation-time constraints we fitted the (sparse) relatedness matrix calculated from a pedigree instead of the (full) ‘genomic’ relatedness matrix. This should not affect the results as results for a simple model testing only the SNP effect (RepeatABEL cannot fit interactions) run in RepeatABEL (genomic relatedness matrix) and ASReml (pedigree relatedness matrix) correlated highly (Supplementary Figure S2).

An assumption underlying linear regression and ANOVA is that the variance of the dependent variable is homoscedastic with respect to the independent variable, i.e., that its variance does not change with the covariate in linear regression or that its variance is roughly equal in the different factor groups in ANOVA. If this assumption is violated, the resulting heteroscedasticity leads to biased results. This problem is exacerbated by fitting SNP^∗^environment interactions and can lead to inflation of *p*-values as has been recognized in the field of psychiatric genetics (Voorman et al., 2011; Almli et al., 2014). To account for this problem we allowed the residual variance to differ among temperature classes in the models fitted with ASReml. Residual variances did indeed differ among temperature classes and fitting them avoided inflating *p*-values. See Supplementary Materials for further details.

Because the standard Bonferroni correction is generally considered overly conservative, an adjusted Bonferroni correction taking linkage disequilibrium (LD) between all SNP loci into account has been suggested (Moskvina and Schmidt, 2008). We calculated this correction with a sliding window of 20 SNPs using the software KEFF VSEP 2007 (Moskvina and Schmidt, 2008). However, because of low LD between SNPs in our great tit population, the amount of effective tests, on which the Bonferroni correction depends, dropped by just 4% and we hence did not apply this correction.

Although previous work in of the study populations analyzed here showed that egg-laying dates are heritable in general (Gienapp et al., 2006; Husby et al., 2010) and also under different spring temperatures (Husby et al., 2011), we here analyzed whether egg-laying date would be heritable in each of the three temperature classes. To increase the power of our analysis, we not only included the genotyped individuals but all females with known identity in our analysis. This data set consisted of 4624 observations of 4032 females, 3737 observations of 3019 females and 4147 of 3532 females for the cold, medium and warm temperature class, respectively. We hence had to include a pedigree-based relatedness matrix instead of a genomic relatedness matrix in our animal model. Besides the additive genetic (random) effect, we fitted female identity as random effect to account for repeated observations as well as age (two level factor as above), year (as factor), area, and the interaction year^∗^area as fixed effects. These analyses were also run in ASReml-R 3.0.

## Results

The heritability of egg-laying date varied among temperature classes. In line with earlier results (Husby et al., 2011) it was lowest under cold spring temperatures (0.14 ± 0.05, estimate and SE) and increased with temperature to 0.38 ± 0.06 and 0.41 ± 0.06 in the medium and warm temperature classes, respectively. The additive genetic variance was statistically significant for all three temperature classes (LRT with 1 df: χ^2^ = 9.39, *p* = 0.002; χ^2^ = 41.9, *p* < 0.001; χ^2^ = 57.5, *p* < 0.001, **Table 1**).

**Table 1:** Variance components (and SE) from quantitative genetic analysis of egg-laying date, separately by temperature class.

	Cold	Medium	Warm
*V*_A_	3.75 ± 1.37	10.8 ± 1.82	9.69 ± 1.41
*h*^2^	0.14 ± 0.05	0.38 ± 0.06	0.41 ± 0.06
*V*_PE_	8.24 ± 1.63	3.00 ± 1.80	2.27 ± 1.43
*V*_res_	15.4 ± 0.90	14.5 ± 0.77	11.9 ± 0.69
No. observations	4624	3737	4147
No. individuals	4032	3019	3532


*Age (‘first year breeder’ vs. ‘older’), area, year (as factor) and the interaction between area and year were included as fixed effects.*

**FIGURE 1:**
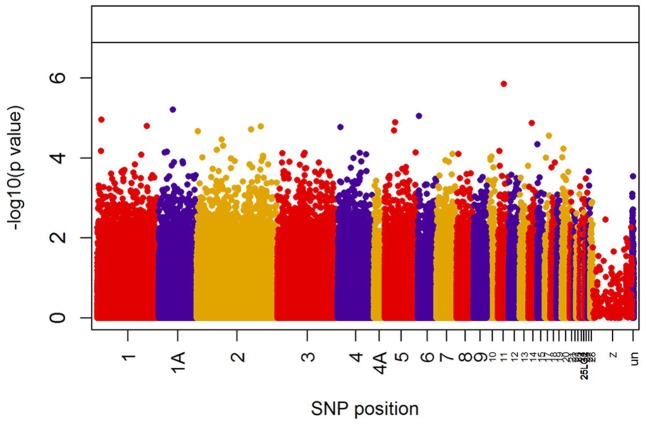
Significances (shown as –log10) of all SNPs included in the genome-wide association analyses of egg-laying date assuming temperature-independent SNP effects. The black line indicates the genome-wide significance level equivalent to *P* = 0.05 after applying a Bonferroni correction. Points are color-coded by chromosome.

**FIGURE 2:**
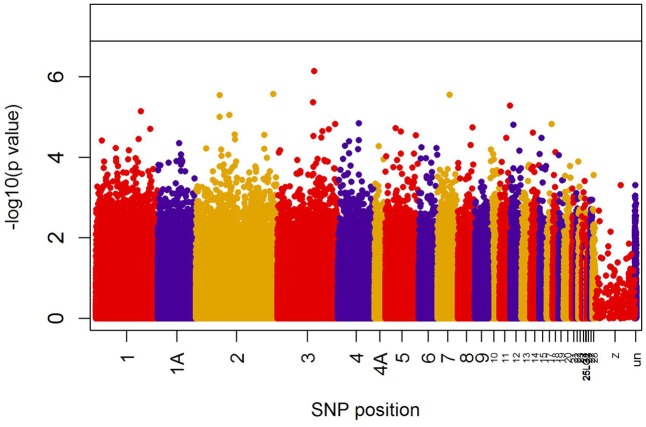
Significances (shown as –log10) of all SNPs included in the genome-wide association analyses of egg-laying date testing for an *interaction* between temperature and SNP effects on laying date. The black line indicates the genome-wide significance level equivalent to *P* = 0.05 after applying a Bonferroni correction. Points are color-coded by chromosome.

**FIGURE 3:**
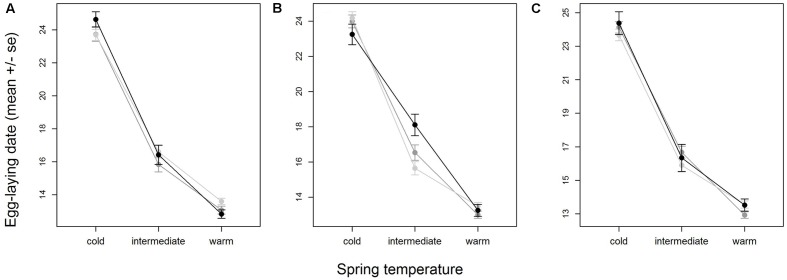
Variation in egg-laying dates of SNP genotypes with temperature for the three SNPs with the most significant interaction with temperature (**Table 2**), AX-100216683 **(A)**, AX-100724221 **(B)** and AX-100140360 **(C)**. Shown are means and standard errors, indicated by whiskers, for homozygote 1 (light gray), heterozygote (medium gray) and homozygote 2 (black). Plotting the area- and year-centered egg-laying dates that were used in the analysis would give a horizontal mean reaction norm. To show the real differences in egg-laying dates with temperature, we here plotted ‘raw’ egg-laying dates that were not corrected for year- and area effects. Note that therefore part of the variation within SNP- and temperature-classes is due to variation among areas. Sample sizes per temperature class-genotype group ranged between 77 and 757 individuals and were on average 381.2.

When all years were analyzed together, no SNP was associated with year- and area-centered egg-laying date at the genome-wide significance level (**Figure 1**). When we tested whether SNP effects differed between cold, intermediate and warm springs, without fitting heterogeneous residuals, two SNPs reached genome-wide significance and one SNP was close to genome-wide significance (Supplementary Table S2). In this model *p*-values were, however, substantially inflated (Supplementary Figure S3A). Fitting heterogeneous residuals in the SNP^∗^temperature interaction model removed this inflation (Supplementary Figure S3B) but also meant that no SNP reached genome-wide significance anymore (**Figure 2** and **Table 2**). The two SNPs that reached genome-wide significance and the SNP that almost reached genome-wide significance were, however, still among the 10 most significant SNPs when heterogeneous residuals were fitted (Supplementary Table S2). Three SNPs that were among the 10 most significant SNPs were located within the thyroglobulin (*TG*) gene, the heparan sulfate-glucosamine 3-sulfotransferase 5 (*HS3ST5*) gene and the teneurin transmembrane protein 4 (*TENM4*) gene, respectively (**Table 2**). For illustration, we plotted mean egg-laying dates against genotypes and spring temperature for these three SNPs (**Figure 3**). In cold springs the birds laid about 10 days later than in warm springs. The maximum difference in egg-laying dates between SNP genotypes was 0.94 days in cold springs, and 0.77 days in warm springs but 2.46 days in intermediate springs. In all three SNPs no genotype bred consistently earlier under all spring temperatures.

**Table 2:** The 10 most significant SNPs for genome-wide association analyses testing for an interaction between SNP effects and temperature, ordered by significance.

SNP	Chr	Genome pos.	*p*-value	MAF	Ref. allele	Gene symbol	Gene name
AX-100216683	3	68133962	7.29*e*-07	0.457	G		
AX-100724221	2	143054586	2.69*e*-06	0.383	G	TG	Thyroglobulin
AX-100140360	7	22714628	2.78*e*-06	0.328	G		
AX-100642627	2	44169247	2.86*e*-06	0.377	C		
AX-100451185	3	66153471	4.29*e*-06	0.179	T	HS3STS	Heparan sulfate-glucosamine 3-sulfotransferase 5
AX-100648937	11	19517225	5.19*e*-06	0.200	T		
AX-100709598	1	83156541	7.15*e*-06	0.337	G	TENM4	Teneurin transmembrane protein 4
AX-100983019	2	61825937	8.91*e*-06	0.270	A		
AX-100667139	2	44170225	1.00*e*-05	0.350	G		
AX-100513411	4	39192295	1.44*e*-05	0.256	C		


*Given are SNP name, on which chromosome it is located (Chr), physical genome position in great tit reference genome (Genome position), *p*-values of GWA analyses, minor allele frequencies (MAF), reference allele and symbols and names of associated genes. Critical *p*-value after Bonferroni correction was 1.30*e-*07. SNPs were assigned to genes based on their genomic position by using the great tit reference annotation (NCBI *Parus major* Annotation Release 101).*

**FIGURE 4:**
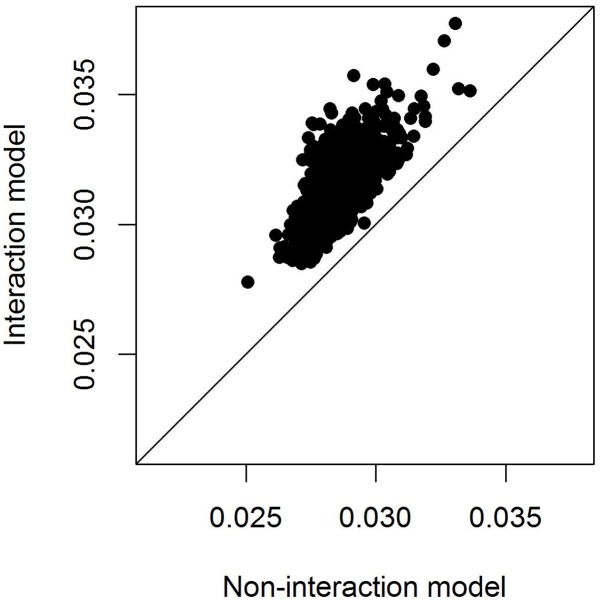
Adjusted marginal *r*^2^ of models with and without SNP^∗^temperature interaction. The marginal *r*^2^ measures the variation explained by fixed effects only and is hence appropriate here because the random effects structure of the models is identical. The *r*^2^ is only plotted for models in which either the SNP effect or the interaction SNP^∗^temperature was significant at the 0.05 level.

To compare model fit of the models with and without SNP^∗^temperature interaction we calculated the marginal *r*^2^ following Nakagawa and Schielzeth (2013), i.e., the variance explained by the fixed effects alone. Even after adjusting for the higher model complexity the model including the SNP^∗^temperature interaction performed consistently better than the model fitting only the SNP effect (**Figure 4**).

## Discussion

Using data from 2045 wild great tits that were genotyped for more than 500k SNPs we could not identify any SNPs that were associated with egg-laying date. However, including an interaction between SNP effects and spring temperature improved model fit (**Figure 4**) although no SNP reached significance after multiple testing correction (**Figure 2**). This result shows that including the effects that the environment has on genotype-phenotype relationship can improve our understanding of this relationship. This has been realized previously in some research fields, as, e.g., animal and plant breeding or psychiatrical genetics (Kraft et al., 2007; e.g., Lindström et al., 2009; Korte and Farlow, 2013; Silva et al., 2014) but not in evolutionary genomics of wild populations.

Fitting an interaction between loci and an environmental variable can only identify loci that are responsible for trait variation in a given environment but not the loci that are responsible for phenotypic plasticity itself. Mapping the genes that are responsible for phenotypic plasticity by altering gene expression or post-translational mechanisms is possible if genetic variation in reaction norm slopes exist and reaction norms of individuals or genotypes, e.g., using clones, can be reliably quantified (e.g., Schadt et al., 2003; Tétard-Jones et al., 2011).

The phenotypic variance in egg-laying dates varied with temperature and to account for this heteroscedasticity we fitted heterogeneous residuals in more SNP^∗^temperature interaction model, i.e., allowed the residual variance to differ among temperature classes. Not doing so would have let to inflated *p*-values because an important assumption of linear models was violated. This is a potential problem in many statistical analysis and may be more common but the *p*-value inflation often goes undetected and becomes only apparent when a (very) large number of tests are performed, as for example in GWAS. The inflation of *p*-values due to heteroscedasticity is likely more problematic when SNP^∗^environment interactions are fitted but could theoretically also occur when only SNP effects are tested. It is normally assumed that variants at a locus affect the mean but they may also affect the variance of the trait (e.g., Ansel et al., 2008; Shen et al., 2012) and thereby lead to heteroscedasticity among genotypes. An observed inflation in *p*-values in GWAS may hence not only be due to population structure, which can be dealt with by applying ‘genomic control’ but also due to heteroscedasticity. In the latter case, however, applying ‘genomic control’ will not be sufficient and fitting heterogeneous residuals, as we did here, or other approaches (Voorman et al., 2011) to correct for this problem are needed.

Among the 10 most significant SNPs whose effects on breeding time differed depending on spring temperature in the year the phenotype was expressed, three were associated with genes of known function. One SNP is located within the thyroglobulin (*TG*) gene. Thyroglobulin is a precursor for thyroid hormones which form an integral part of the Hypothalamus–pituitary–thyroid axis, which has an important role in controlling general metabolism (Dietrich et al., 2012). In bulls the genetic variance in *TG* has been linked to age of puberty (Fernández et al., 2014) and thyroid hormones affect reproduction in both sexes (Flood et al., 2013). Another SNP was located within the heparan sulfate-glucosamine 3-sulfotransferase 5 (*HS3ST5*) gene, which is involved in heparan sulfate biosynthesis and glycosaminoglycan metabolism. Heparan sulfate is involved in cell-signaling and has various essential functions in development and homeostasis (Li and Kusche-Gullberg, 2016). Like for *HS3ST5*, Glycosaminoglycans are involved in cell-signaling and have structural functions in connective tissue, bone and blood vessels (Esko et al., 2009). The third SNP was located within the teneurin transmembrane protein 4 (*TENM4*). Teneurins are a highly conserved gene family that function as cell surface signal molecules and transcriptional regulators (Tucker and Chiquet-Ehrismann, 2006). *TENM4* has been shown to play a role in neural development in chicken (Tucker et al., 2000).

Genome-wide association studies have worked well in certain fields, e.g., in case-control studies of human diseases (Visscher et al., 2012) but identifying genes underlying quantitative traits, especially in natural populations has proven to be much more difficult. Besides other reasons, as, e.g., unknown environmental effects on phenotypes that could not be accounted for, limited power or overly conservative multiple testing corrections, one potential reason for this is variation in phenotypes due to phenotypic plasticity. This variation can be accounted for if the environmental driver of the plasticity or the temporal or spatial scale on that it varies is known. For example, egg-laying dates vary strongly from year-to-year driven by spring temperatures. We can therefore account for this plasticity by correcting phenotypes for annual means; even if we had no idea which environmental variable was driving the annual variation. Phenotypic plasticity can, however, also ‘obscure’ genotype-phenotype associations because different loci are associated with the trait in different environments due to differential gene expression levels or post-translational mechanisms. An important aspect when testing whether genotype-phenotype associations vary across environments is obviously to identify the correct environmental variable that drives plastic variation in the trait. We here used spring temperature from March 11 to April 20, which has been shown to predict egg-laying dates fairly well in our study population (Husby et al., 2011), to classify our environment. However, using two temperature classes instead of three led to different results, in terms of the SNPs included in the best models. This highlights the problem of identifying a meaningful environmental variable when fitting SNP^∗^environment interactions.

Most traits are phenotypically plastic (Pigliucci, 2001) and the lack of awareness of the variable genetic architecture underlying plastic traits may explain why identifying genes underlying quantitative traits has proven to be a major challenge. We here found indication for such variable SNP effects in plastic traits. This demonstrates that explicitly modeling phenotypic plasticity can be crucial for genome-wide association studies, especially – but not only – in studies of wild populations, which would thereby contribute to our still limited knowledge of the genome-phenome link in ecologically relevant traits, which is especially important as species need to adapt to their changing world.

## Author Contributions

PG and MV compiled the phenotypic data. VL compiled the genomic data. PG and VL conducted all analyses. AM organized the DNA samples and extracted the DNA. PG, VL, KvO, and MV developed this research, PG wrote the manuscript with contributions from VL and all co-authors commented on it.

## Conflict of Interest Statement

The authors declare that the research was conducted in the absence of any commercial or financial relationships that could be construed as a potential conflict of interest.

## Footnotes

1 https://data.knmi.nl/datasets
