# Supplementary material for: Environment-Dependent Genotype-Phenotype Associations in Avian Breeding Time
Source: Front Genet. 2017 Aug 4;8:102. doi: 10.3389/fgene.2017.00102 (PMC5543038; doi:10.3389/fgene.2017.00102)
Supplement: Supplementary file 1 [file Table_1.DOCX]

***Supplementary Material***

**Environment-Dependent Genotype-Phenotype Associations in Avian Breeding Time**

**Phillip Gienapp*, Veronika N. Laine, A. Christa Mateman, Kees van Oers, Marcel E. Visser**

*** Correspondence:** Phillip Gienapp, p.gienapp@nioo.knaw.nl

**Table S1**. Overview over QTL mapping studies in wild populations with information on taxon, species, analyzed trait, trait class (LH: life history, M: morphology, P: physiology, B: behavior and O: other), method used (AM: association mapping, PLM: pedigree linkage mapping), number of individuals and markers (SNP: single nucleotide polymorphism, MS: micro-satellite, AFLP: amplified fragment length polymorphism, Alloz: allozyme), number of loci found, and the percentage in additive genetic (*V*_A_) and/or total phenotypic variation (*V_P_*) explained by the identified loci. Note that if several studies analyzed the same trait in the same population (using different approaches or numbers of markers), all studies are listed here.

| Taxon | Species | Trait | trait class | Method | No. individuals | No. markers | no. loci found | *V_A_* / *V_P_* explained | Reference |
| --- | --- | --- | --- | --- | --- | --- | --- | --- | --- |
| plant | Lodgepole pine (*Pinus contorta*) | cone serotiny | LH | AM ^(1)^ | 98 | 97616 SNP | 11 | 50% of *V_P_* | ([Parchman et al., 2012](#_ENREF_12)) |
| plant | Maritime pine (*Pinus pinaster*) | serotiny | LH | AM | 199 | 384 SNP | 17^(3)^ | 29% of *V_P_* ^(6)^ | ([Budde et al., 2014](#_ENREF_6)) |
| plant | Loblolly pine (*Pinus taeda*) | tree height | M | AM ^(2)^ | 380 | 3938 SNP | 1 | - | ([Cumbie et al., 2011](#_ENREF_7)) |
| plant | Loblolly pine (*Pinus taeda*) | carbon isotope discrimination | P | AM ^(2)^ | 380 | 3938 SNP | 4 / 7 ^(4)^ | - | ([Cumbie et al., 2011](#_ENREF_7)) |
| plant | Loblolly pine (*Pinus taeda*) | foliar nitrogen concentration | P | AM ^(2)^ | 380 | 3938 SNP | 5 / 6 ^(4)^ | - | ([Cumbie et al., 2011](#_ENREF_7)) |
| fish | Rainbow trout (*Oncorhynchus mykiss*) | migration propensity | LH | AM | 182 | 5019 SNP | 58 | - | ([Hecht et al., 2013](#_ENREF_8)) |
| fish | Salmon (*Salmo salar*) | sea age | LH | AM | 1518 | 167410 / 208704 SNP | 1 region with two candidate genes | 39% of *V_P_* | ([Barson et al., 2015](#_ENREF_1)) |
| fish | Rainbow trout (*Oncorhynchus mykiss*) | ATPase activity | P | AM | 127 | 6039 SNP | 49 | - | ([Hecht et al., 2013](#_ENREF_8)) |
| bird | Collared flycatcher (*Ficedula albicollis*) | clutch size | LH | AM | 313 | 37 309 SNP | 1 | 4% of *V_P_* | ([Husby et al., 2015](#_ENREF_9)) |
| bird | Great tit (*Parus major*) | egg mass | LH | PLM, AM | 902 | 7203 SNP | 0 | - | ([Santure et al., 2013](#_ENREF_16)) |
| bird | Great tit (*Parus major*) | clutch size | LH | PLM, AM | 969 | 7203 SNP | 0 | - | ([Santure et al., 2013](#_ENREF_16)) |
| bird | Great tit (*Parus major*) | egg mass (UK) | LH | PLM, AM | 678 / 960 | 5591 SNP | 0 | - | ([Santure et al., 2015](#_ENREF_15)) |
| bird | Great tit (*Parus major*) | clutch size (UK) | LH | PLM, AM | 722 / 1026 | 5591 SNP | 0 | - | ([Santure et al., 2015](#_ENREF_15)) |
| bird | Great tit (*Parus major*) | fledgling weight (as maternal trait) (UK) | LH | PLM, AM | 328 / 441 | 5591 SNP | 0 | - | ([Santure et al., 2015](#_ENREF_15)) |
| bird | Great tit (*Parus major*) | adult weight (UK) | M | PLM, AM | 1360 / 1872 | 5591 SNP | 0 | - | ([Santure et al., 2015](#_ENREF_15)) |
| bird | Great tit (*Parus major*) | fledgling weight (of individual) (UK) | M | PLM, AM | 1183 / 1222 | 5591 SNP | 0 | - | ([Santure et al., 2015](#_ENREF_15)) |
| bird | Great tit (*Parus major*) | tarsus length (UK) | M | PLM, AM | 626 / 872 | 5591 SNP | 0 | - | ([Santure et al., 2015](#_ENREF_15)) |
| bird | Great tit (*Parus major*) | wing length (UK) | M | PLM, AM | 1410 / 1949 | 5591 SNP | 0 | - | ([Santure et al., 2015](#_ENREF_15)) |
| bird | Great tit (*Parus major*) | exploratory behaviour (UK) | B | PLM, AM | 743 / 1046 | 5591 SNP | 0 | - | ([Santure et al., 2015](#_ENREF_15)) |
| bird | Great tit (*Parus major*) | clutch size (NL) | LH | PLM, AM | 403 / 943 | 5591 SNP | 0 | - | ([Santure et al., 2015](#_ENREF_15)) |
| bird | Great tit (*Parus major*) | fledgling weight (as maternal trait) (NL) | LH | PLM, AM | 327 / 744 | 5591 SNP | 0 | - | ([Santure et al., 2015](#_ENREF_15)) |
| bird | Great tit (*Parus major*) | adult weight (NL) | M | PLM, AM | 408 / 477 | 5591 SNP | 0 | - | ([Santure et al., 2015](#_ENREF_15)) |
| bird | Great tit (*Parus major*) | fledgling weight (of individual) (NL) | M | PLM, AM | 357 / 416 | 5591 SNP | 0 | - | ([Santure et al., 2015](#_ENREF_15)) |
| bird | Great tit (*Parus major*) | tarsus length (NL) | M | PLM, AM | 653 / 1378 | 5591 SNP | 0 | - | ([Santure et al., 2015](#_ENREF_15)) |
| bird | Great tit (*Parus major*) | wing length (NL) | M | PLM, AM | 590 / 1275 | 5591 SNP | 0 | - | ([Santure et al., 2015](#_ENREF_15)) |
| bird | Great tit (*Parus major*) | exploratory behaviour (NL) | B | PLM, AM | 462 / 912 | 5591 SNP | 0 | - | ([Santure et al., 2015](#_ENREF_15)) |
| bird | Red grouse (*Lagopus lagopus*) | parasite load | LH | AM | 649-695 | 384 SNP | 5 | 5% of *V_P_* | ([Wenzel et al., 2015](#_ENREF_19)) |
| bird | Great reed warbler (*Acrocephalus arundinaceus*) | tarsus length | M | PLM | 333 | 57 MS, 36 AFLP | 0 | - | ([Tarka et al., 2010](#_ENREF_18)) |
| bird | Great reed warbler (*Acrocephalus arundinaceus*) | wing length | M | PLM | 333 | 57 MS, 36 AFLP | 1 ^(5)^ | 37% of *V_P_*, 55% of *V_A_* | ([Tarka et al., 2010](#_ENREF_18)) |
| mammal | Bighorn sheep (*Ovis canadensis*) | boldness | B | PLM | 137 | 238 MS | 0 | - | ([Poissant et al., 2013](#_ENREF_14)) |
| mammal | Bighorn sheep (*Ovis canadensis*) | docility | B | PLM | 77 | 238 MS | 0 | - | ([Poissant et al., 2013](#_ENREF_14)) |
| mammal | Red deer (*Cervus elaphus*) | birth weight | LH | PLM | 295 | 90 MS | 0 | 100% of *V_A_*(?) | ([Slate et al., 2002](#_ENREF_17)) |
| mammal | Soay sheep (*Ovis aries*) | birth date | LH | PLM | 526 | 247 MS, 4 Alloz | 0 | - | ([Beraldi et al., 2007b](#_ENREF_4)) |
| mammal | Soay sheep (*Ovis aries*) | birth date (as maternal trait) | LH | PLM | 136 | 247 MS, 4 Alloz | 0 | - | ([Beraldi et al., 2007b](#_ENREF_4)) |
| mammal | Soay sheep (*Ovis aries*) | birth weight | LH | PLM | 507 | 247 MS, 4 Alloz | 0 | - | ([Beraldi et al., 2007b](#_ENREF_4)) |
| mammal | Soay sheep (*Ovis aries*) | birth weight (as maternal trait) | LH | PLM | 133 | 247 MS, 4 Alloz | 0 | - | ([Beraldi et al., 2007b](#_ENREF_4)) |
| mammal | Soay sheep (*Ovis aries*) | foreleg length | M | PLM | 509 | 247 MS, 4 Alloz | 0 | - | ([Beraldi et al., 2007b](#_ENREF_4)) |
| mammal | Soay sheep (*Ovis aries*) | foreleg length (adults) | M | PLM | 436 | 247 MS, 4 Alloz | 0 | - | ([Beraldi et al., 2007b](#_ENREF_4)) |
| mammal | Soay sheep (*Ovis aries*) | hindleg length | M | PLM | 512 | 247 MS, 4 Alloz | 0 | - | ([Beraldi et al., 2007b](#_ENREF_4)) |
| mammal | Soay sheep (*Ovis aries*) | hindleg length (adults) | M | PLM | 436 | 247 MS, 4 Alloz | 0 | - | ([Beraldi et al., 2007b](#_ENREF_4)) |
| mammal | Soay sheep (*Ovis aries*) | body weight | M | PLM | 407 | 247 MS, 4 Alloz | 0 | - | ([Beraldi et al., 2007b](#_ENREF_4)) |
| mammal | Soay sheep (*Ovis aries*) | body weight (adults) | M | PLM | 228 | 247 MS, 4 Alloz | 0 | - | ([Beraldi et al., 2007b](#_ENREF_4)) |
| mammal | Soay sheep (*Ovis aries*) | metacarpal length | M | PLM | 332 | 247 MS, 4 Alloz | 0 | - | ([Beraldi et al., 2007b](#_ENREF_4)) |
| mammal | Soay sheep (*Ovis aries*) | jaw length | M | PLM | 396 | 247 MS, 4 Alloz | 0 | - | ([Beraldi et al., 2007b](#_ENREF_4)) |
| mammal | Soay sheep (*Ovis aries*) | pathogen resistance | LH | PLM | 228-396 | 247 MS / 4 Alloz | 0 | - | ([Beraldi et al., 2007a](#_ENREF_2)) |
| mammal | Bighorn sheep (*Ovis canadensis*) | body mass | M | PLM | 310 | 247 MS | 0 | - | ([Poissant et al., 2012](#_ENREF_13)) |
| mammal | Bighorn sheep (*Ovis canadensis*) | horn volume | M | PLM | 310 | 247 MS | 0 | - | ([Poissant et al., 2012](#_ENREF_13)) |
| mammal | Bighorn sheep (*Ovis canadensis*) | horn length | M | PLM | 310 | 247 MS | 0 | - | ([Poissant et al., 2012](#_ENREF_13)) |
| mammal | Bighorn sheep (*Ovis canadensis*) | horn base circumference | M | PLM | 310 | 247 MS | 0 | - | ([Poissant et al., 2012](#_ENREF_13)) |
| mammal | Soay sheep (*Ovis aries*) | coat colour | M | PLM | 560 | 247 MS / 4 Alloz | 1 | - | ([Beraldi et al., 2006](#_ENREF_3)) |
| mammal | Soay sheep (*Ovis aries*) | coat pattern | M | PLM | 560 | 247 MS / 4 Alloz | 1 | - | ([Beraldi et al., 2006](#_ENREF_3)) |
| mammal | Soay sheep (*Ovis aries*) | horn type | M | PLM | 560 | 247 MS / 4 Alloz | 1 | - | ([Beraldi et al., 2006](#_ENREF_3)) |
| mammal | Soay sheep (*Ovis aries*) | metacarpal length | M | PLM | 588 | 247 MS / 4 Alloz | 0 | - | ([Beraldi et al., 2007b](#_ENREF_4)) |
| mammal | Soay sheep (*Ovis aries*) | jaw length | M | AM | ~900 | 37037 SNP | 0 | - | ([Bérénos et al., 2015](#_ENREF_5)) |
| mammal | Soay sheep (*Ovis aries*) | foreleg length | M | AM | ~900 | 37037 SNP | 0 | - | ([Bérénos et al., 2015](#_ENREF_5)) |
| mammal | Soay sheep (*Ovis aries*) | weight | M | AM | ~900 | 37037 SNP | 0 | - | ([Bérénos et al., 2015](#_ENREF_5)) |
| mammal | Soay sheep (*Ovis aries*) | metacarpal length | M | AM | ~900 | 37037 SNP | 4 |  | ([Bérénos et al., 2015](#_ENREF_5)) |
| mammal | Soay sheep (*Ovis aries*) | horn size | M | AM | 160 | 35831 SNP | several |  | ([Johnston et al., 2011](#_ENREF_11)) |
| mammal | Soay sheep (*Ovis aries*) | horn type | M | AM | 445 | 35831 SNP | 1 region |  | ([Johnston et al., 2011](#_ENREF_11)) |
| mammal | Soay sheep (*Ovis aries*) | recombination rate | O | AM | 3330 | 39104 SNP | 2 regions | 73% of *V_A_* in females, 26% of *V_A_* in males | ([Johnston et al., 2016](#_ENREF_10)) |

(1) based on selection of extreme phenotypes; (2) in common garden setting; (3) unclear whether significant after proper multiple testing correction; (4) depending on model; (5) covering seven markers; (6) on chip, unclear how much retained for analysis; (7) estimated from separate model

**References in Table S1**

Barson, N.J., Aykanat, T., Hindar, K., Baranski, M., Bolstad, G.H., Fiske, P., et al. (2015). Sex-dependent dominance at a single locus maintains variation in age at maturity in salmon. *Nature* 528**,** 405-408.

Beraldi, D., McRae, A.F., Gratten, J., Pilkington, J.G., Slate, J., Visscher, P.M., et al. (2007a). Quantitative trait loci (QTL) mapping of resistance to strongyles and coccidia in the free-living Soay sheep (*Ovis aries*). *Int. J. Parasitol.* 37**,** 121-129.

Beraldi, D., McRae, A.F., Gratten, J., Slate, J., Visscher, P.M., and Pemberton, J.M. (2006). Development of a linkage map and mapping of phenotypic polymorphisms in a free-living population of Soay sheep (*Ovis aries*). *Genetics* 173**,** 1521-1537.

Beraldi, D., McRae, A.F., Gratten, J., Slate, J., Visscher, P.M., and Pemberton, J.M. (2007b). Mapping quantitative trait loci underlying fitness-related traits in a free-living sheep population. *Evolution* 61**,** 1403-1416.

Bérénos, C., Ellis, P.A., Pilkington, J.G., Lee, S.H., Gratten, J., and Pemberton, J.M. (2015). Heterogeneity of genetic architecture of body size traits in a free-living population. *Mol. Ecol.* 24**,** 1810-1830.

Budde, K.B., Heuertz, M., Hernández-Serrano, A., Pausas, J.G., Vendramin, G.G., Verdú, M., et al. (2014). *In situ* genetic association for serotiny, a fire-related trait, in Mediterranean maritime pine (*Pinus pinaster*). *New Phytol.* 201.

Cumbie, W.P., Eckert, A., Wegrzyn, J., Whetten, R., Neale, D., and Goldfarb, B. (2011). Association genetics of carbon isotope discrimination, height and foliar nitrogen in a natural population of *Pinus taeda* L. *Heredity* 107**,** 105-114.

Hecht, B.C., Campbell, N.R., Holeck, D.E., and Narum, S.R. (2013). Genome-wide association reveals genetic basis for the propensity to migrate in wild populations of rainbow and steelhead trout. *Mol. Ecol.* 22**,** 3061-3076.

Husby, A., Kawakami, T., Rönnegård, L., Smeds, L., Ellegren, H., and Qvarnström, A. (2015). Genome-wide association mapping in a wild avian population identifies a link between genetic and phenotypic variation in a life-history trait. *Proc. R. Soc. B* 282**,** 20150156.

Johnston, S.E., Bérénos, C., Slate, J., and Pemberton, J.M. (2016). Conserved genetic architecture underlying individual recombination rate variation in a wild population of Soay sheep (*Ovis aries*). *Genetics* 203**,** 583-598.

Johnston, S.E., McEwan, J.C., Pickering, N.K., Kijas, J.W., Beraldi, D., Pilkington, J.G., et al. (2011). Genome-wide association mapping identifies the genetic basis of discrete and quantitative variation in sexual weaponry in a wild sheep population. *Mol. Ecol.* 20**,** 2555-2566.

Parchman, T.L., Gompert, Z., Mudge, J., Schilkey, F.D., Benkman, C.W., and Buerkle, C.A. (2012). Genome-wide association genetics of an adaptive trait in lodgepole pine. *Mol. Ecol.* 21**,** 2991-3005.

Poissant, J., Davis, C.S., Malenfant, R.M., Hogg, J.T., and Coltman, D.W. (2012). QTL mapping for sexually dimorphic fitness-related traits in wild bighorn sheep. *Heredity* 108**,** 256-263.

Poissant, J., Réale, D., Martin, J.G.A., Festa-Bianchet, M., and Coltman, D.W. (2013). A quantitative trait locus analysis of personality in wild bighorn sheep. *Ecol. Evol.* 3**,** 474-481.

Santure, A.E., Poissant, J., De Cauwer, I., van Oers, K., Robinson, M.R., Quinn, J.L., et al. (2015). Replicated analysis of the genetic architecture of quantitative traits in two wild great tit populations. *Mol. Ecol.* 24**,** 6148-6162.

Santure, A.W., De Cauwer, I., Robinson, M.R., Poissant, J., Sheldon, B.C., and Slate, J. (2013). Genomic dissection of variation in clutch size and egg mass in a wild great tit (*Parus major*) population. *Mol. Ecol.* 22**,** 3949-3962.

Slate, J., Visscher, P.M., MacGregor, S., Stevens, D., Tate, M.L., and Pemberton, J.M. (2002). A genome scan for quantitative trait loci in a wild population of red deer (*Cervus elaphus*). *Genetics* 162**,** 1863-1873.

Tarka, M., Åkesson, M., Beraldi, D., Hernández-Sánchez, J., Hasselquist, D., Bensch, S., et al. (2010). A strong quantitative trait locus for wing length on chromosome 2 in a wild population of great reed warblers. *Proc. R. Soc. B* 277**,** 2361-2369.

Wenzel, M.A., James, M.C., Douglas, A., and Piertney, S.B. (2015). Genome-wide association and genome partitioning reveal novel genomic regions underlying variation in gastrointestinal nematode burden in a wild bird. *Mol. Ecol.* 24**,** 4175-4192.

**Table S2**. Ten most significant SNPs for genome-wide association analyses fitting an interaction between SNP and temperature but no heterogeneous residual variance, ordered by significance. Given are SNP name, on which chromosome it is located (Chr), physical genome position in great tit reference genome (Genome position), *p*-values of GWA analyses, minor allele frequencies (MAF) and reference allele. The critical *p*-value after Bonferroni correction is 1.17e-07, which means that the first two were genome-wide significant and the third almost so. P-values from these models were, however, inflated due to heteroscedasticity.

| SNP | Chr | Genome pos. | *p*-value | MAF | Ref. allele |
| --- | --- | --- | --- | --- | --- |
| AX-100451185 | 3 | 66153471 | 5.16e-08 | 0.179 | T |
| AX-100724221 | 1 | 143054586 | 5.26e-08 | 0.383 | G |
| AX-100709598 | 1 | 83156541 | 1.43e-07 | 0.337 | G |
| AX-100642627 | 2 | 44169247 | 2.66e-07 | 0.377 | C |
| AX.100385858 | 3 | 66829287 | 1.04e-06 | 0.266 | A |
| AX.100983019 | 2 | 61825937 | 1.39e-06 | 0.270 | A |
| AX.100648937 | 11 | 19517225 | 1.49e-06 | 0.200 | T |
| AX.100127958 | 8 | 26833266 | 1.67e-06 | 0.370 | T |
| AX.100133629 | 3 | 2776790 | 1.67e-06 | 0.278 | A |
| AX-100783468 | 18 | 6022144 | 1.79e-06 | 0.190 | G |

**Fig. S1**. Multi-dimensional scaling (MDS) plot of pairwise IBS distances. Using PLINK, pairwise IBS was calculated for all SNPs included in the analysis (see Methods for inclusion criteria of SNPs), converted to a distance matrix and the first two coordinates of the MDS extracted for plotting. Symbols are colour-coded by the temperature class in which an individual bred (red=warm, black=medium, blue=cold). No clear grouping in relation to temperature class is discernible. The filled symbols indicate 21 individuals that were defined as outliers during quality control and excluded from further analysis.


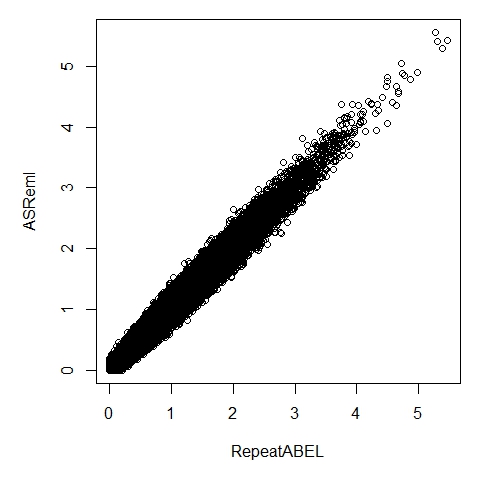


**Fig. S2**. Correlation between results from RepeatABEL and ASReml. The negative Log_10_-values for each SNP from ASreml (using a pedigree-based relatedness matrix) are plotted against the corresponding values from RepeatABEL (using a genomic relatedness matrix). The correlation is 0.99 (t = 3939.3, df = 384081, p-value < 0.001). As interactions between SNP and other variables cannot be fitted in RepeatABEL this results are for the model without SNP*temperature interaction.


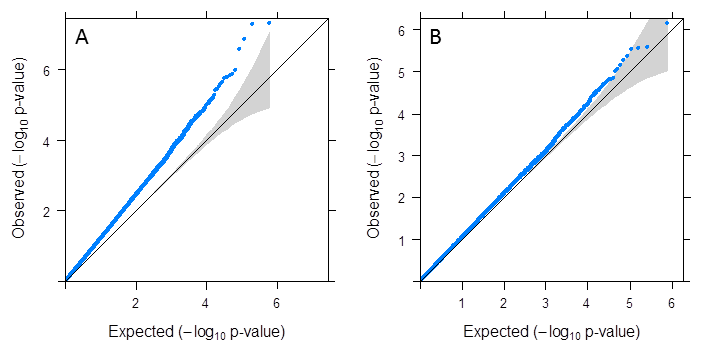


**Fig. S3**. QQ-plots for GWAA testing for SNP*temperature interaction fitting no heterogeneous residual variance (A) and fitting heterogeneous residual variance (B). While fitting the SNP*temperature interaction without heterogeneous residual variance led to p-value inflation (lambda = 1.09), fitting heterogeneous residual variance removed this inflation (lambda = 1.04).
